# Injecting Sustainability into Epoxy-Based Composite Materials by Using Bio-Binder from Hydrothermal Liquefaction Processing of Microalgae

**DOI:** 10.3390/molecules29153656

**Published:** 2024-08-01

**Authors:** Philip Agbo, Abhijeet Mali, Ajit D. Kelkar, Lijun Wang, Lifeng Zhang

**Affiliations:** 1 Department of Nanoengineering, Joint School of Nanoscience and Nanoengineering, North Carolina A&T State University, 2907 E Gate City Blvd., Greensboro, NC 27401, USA; 2 Department of Mechanical Engineering, College of Engineering, North Carolina A&T State University, 1601 E Market St., Greensboro, NC 27411, USA; kelkar@ncat.edu; 3 Department of Natural Resources and Environmental Design, College of Agriculture and Environmental Sciences, North Carolina A&T State University, 1601 E Market St., Greensboro, NC 27411, USA

**Keywords:** microalgae, epoxy resin, hydrothermal liquefaction, sustainability, composite

## Abstract

We report a transformative epoxy system with a microalgae-derived bio-binder from hydrothermal liquefaction processing (HTL). The obtained bio-binder not only served as a curing agent for conventional epoxy resin (e.g., EPON 862), but also acted as a modifying agent to enhance the thermal and mechanical properties of the conventional epoxy resin. This game-changing epoxy/bio-binder system outperformed the conventional epoxy/hardener system in thermal stability and mechanical properties. Compared to the commercial EPON 862/EPIKURE W epoxy product, our epoxy/bio-binder system (35 wt.% bio-binder addition with respect to the epoxy) increased the temperature of 60% weight loss from 394 °C to 428 °C and the temperature of maximum decomposition rate from 382 °C to 413 °C, while the tensile, flexural, and impact performance of the cured epoxy improved in all cases by up to 64%. Our research could significantly impact the USD 38.2 billion global market of the epoxy-related industry by not only providing better thermal and mechanical performance of epoxy-based composite materials, but also simultaneously reducing the carbon footprint from the epoxy industry and relieving waste epoxy pollution.

## 1. Introduction

Epoxy resin is a class of thermosetting pre-polymers that possesses excellent physical properties, such as good processability, excellent mechanical strength, great chemical resistance, high adhesion strength, superior thermal stability, and outstanding insulation capability after curing [1]. Epoxy resin is extensively used in a wide range of commercial products, including coatings, adhesives, and particularly polymer composite materials, in applications such as transportation, construction, aerospace, electronics, etc. Epoxy resin is generally derived from fossil fuel and produced through a series of chemical reactions. The most popular epoxy resin is diglycidyl ether of bisphenol A (DGEBA), which constitutes more than 75% of the epoxy market [2]. DGEBA is produced by a condensation reaction between bisphenol A and epichlorohydrin with a catalyst such as sodium hydroxide [3]. Epoxy resin must go through a curing process to solidify and achieve its final mechanical properties. In the curing process, a chemical compound termed as a curing agent or hardener with two or more functional groups (e.g., -NH_2_) that can react with the epoxide functional groups (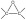) in epoxy resin is added and reacts with epoxy resin at an elevated temperature, forming a 3D crosslinked molecular network. It is noteworthy that the production of conventional epoxy resin and its curing agent requires a large amount of fossil fuel that can significantly contribute to greenhouse gas emissions and climate change. Furthermore, the epoxy products are not biodegradable, and they can stay in our environment for hundreds of years without significant degradation, posing a risk of contamination to air, soil, and water.

Sustainable or green epoxy resin has attracted significant attention in recent years [4,5], and has emerged not only in scientific publications [6,7], but also in real market such as Eco Green Resin from A. C. Composite Resins (Sacramento, CA, USA), Entropy Resin from Entropy Resins Inc. (Bay City, MI, USA), and Ecopoxy Resin from EcoPoxy Inc. (Morris, MB, Canada). Although the sources of these epoxy products are from biomass and sustainable, such as sugar [8], vegetable oil [9], and fatty acid [10], there are a few common limitations, including a requirement of a paired conventional curing agent, high cost of the epoxy resin, high viscosity of the epoxy resin in processing [11], and particularly low mechanical and thermal performance of the cured products compared to their conventional epoxy counterparts [12,13], which can be ascribed to the relatively flexible molecular backbone in those epoxy molecules.

Our research targeted the development of a sustainable epoxy system from renewable resources with high mechanical performance. It is known that algae are a renewable bioresource because of their high photosynthetic efficiency and high growth rates. It is reported that algae can be converted to bio-oil using a hydrothermal liquefaction (HTL) process in which algal biomass directly decomposes in water at a moderate temperature and pressure level [14,15]. The algal bio-oil has been described as similar to conventional petroleum and can be upgraded and used as transportation fuel [16]. However, the bio-oil contains a high nitrogen content, which derives from the protein component of algae. The nitrogen content imparts several undesirable properties for fuel purposes and needs to be reduced [17]. It is noteworthy that the nitrogen content in the bio-oil, such as amine (-NH_2_) functional groups, could react with conventional epoxy resins just like their hardeners. In this research, we acquired a bio-oil from *Chlorella vulgaris*, a single-celled freshwater green microalga that grows naturally in lakes and ponds worldwide, through HTL processing and placed the bio-oil into a brand-new field, i.e., epoxy resin and composite materials. To differentiate its use from biofuel uses, we use the term bio-binder herein to refer to the bio-oil from HTL processing of microalgae. In this research, we incorporated the bio-binder into a representative conventional epoxy resin (EPON 862), investigated the thermal stability and mechanical properties of the epoxy/bio-binder system, and compared the results with those of EPON 862 with a paired hardener (EPICURE W). Our results indicated that the integration of the bio-binder from HTL processing of microalgae with conventional epoxy resin is a game-changing way to inject sustainability into current epoxy-based commercial products, aiming towards future environmentally friendly and high-performance epoxy resin-based composite materials.

## 2. Results and Discussion

### 2.1. Composition and Curing

**Figure 1:**
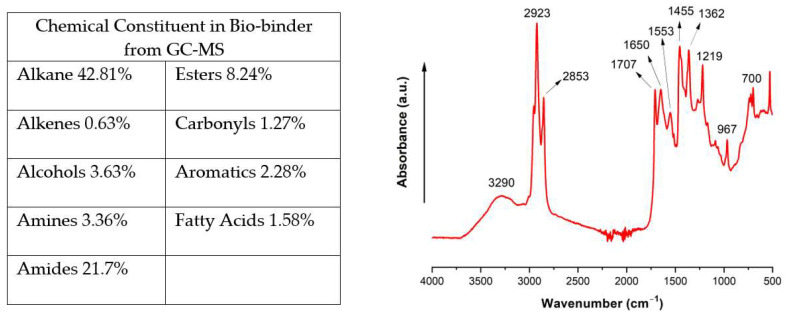
Chemical constituents from GC-MS (area percentage) and FTIR spectra of the bio-binder from HTL processing of the green microalgae.

To learn the composition of the bio-binder obtained from HTL processing of the green microalgae, we performed GC-MS and FTIR analysis (Figure 1). GC-MS analysis indicated that the bio-binder was a complicated mixture of alkanes, amides, esters, alcohols, amines, etc., as primary constituents. The characteristic FTIR spectrum of the bio-binder indicated certain functional groups that were consistent with the GC-MS analysis results. Specifically, the broad IR peak in the range of 3200 cm^−1^ to 3700 cm^−1^ could be attributed to mixed stretching vibrations involving both O-H and N-H bonds. The IR peaks in the region of 3000 cm^−1^ and 3200 cm^−1^ could be assigned to C-H stretching from the aromatic ring and alkene, while the peaks in the region of 2800 cm^−1^ to 3000 cm^−1^ represented C-H stretching vibrations in alkane. The IR peaks centered at ~1707 cm^−1^, ~1650 cm^−1^, and ~1553 cm^−1^ indicated C=O stretching in carboxylic acid, C=O stretching in amide (Amide I)/N-H bending, and C-N stretching/N-H bending (Amide II) along with aromatic ring stretching (C=C-C), respectively. The IR peaks around 1454 cm^−1^ and 1362 cm^−1^ could be assigned to C-H bending in alkane. The IR peak at 1219 cm^−1^ could be ascribed to aromatic and aliphatic C-O/C-N stretching. The IR peaks centered at 968 cm^−1^ and 700 cm^−1^ could represent C=C bending and C-H out-of-plane bending in the benzene ring. All these FTIR peaks indicated the presence of alkane, alkene, and aromatic compounds with hydroxyl, amide, amine, and carboxylic acid functional groups, which can react with the epoxide functional groups in epoxy resin [18].

Accordingly, we studied the composition of the epoxy/bio-binder systems with EPON 862, a popular conventional epoxy for composite use. It is well known that the curing cycle of epoxy resin plays a key role in achieving its desired mechanical property and long-term durability. Following the recommended curing cycle of EPON 862 from the manufacturer, we realized a practicable epoxy system with bio-binder compositions at 30 wt.%, 35 wt.%, and 40 wt.% of EPON 862. With extremely low percentages of the bio-binder, like 10 wt.% and 20 wt.% of the epoxy, the product is not usable (sticky). With high percentages of the bio-binder, like 45 wt.% and 50 wt.% of epoxy, the product is also not usable (too soft and cannot maintain integrity in mechanical tests).

**Figure 2:**
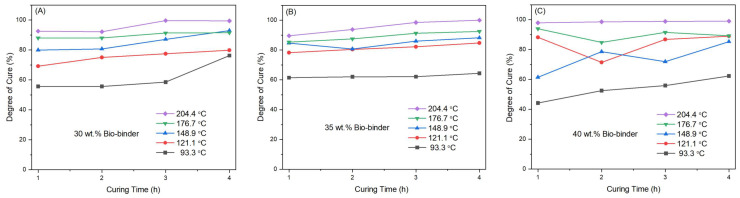
Degree of cure of the epoxy/bio-binder samples at bio-binder loadings of 30 wt.% (**A**), 35 wt.% (**B**), and 40 wt.% (**C**) of epoxy under different curing conditions.

Then, we used DSC to determine the optimal curing cycle for the epoxy/bio-binder system (Figure 2). Based on the recommended curing cycle of EPON 862, we studied five curing temperatures for our epoxy/bio-binder system, i.e., 200 °F, 250 °F, 300 °F, 350 °F, and 400 °F (93.3 °C, 121.1 °C, 148.9 °C, 176.7 °C, and 204.4 °C, correspondingly) and four different curing times, i.e., 1 h, 2 h, 3 h, and 4 h. The degree of cure of each sample was determined by using Equation (1) that took into account the total curing heat and the individual sample’s curing heat in the curing process through DSC [19].

(1) $$\%\;\mathrm{Cure}=\left(1-\frac{DHR}{DHT}\right)\times 100$$

where DHR represents the heat of cure calculated from the area under the curing exothermic peak of the DSC curve of each individual cured epoxy/bio-binder sample, while DHT represents the total heat of cure calculated from the total area under the curing exothermic peak of the DSC curve of the correspondingly uncured epoxy/bio-binder sample. Across our epoxy/bio-binder system, the degree of cure increased with both the curing time and the temperature. The epoxy/bio-binder samples cured at 204.4 °C for 4 h exhibited the highest degree of cure, nearly reaching 100%. In the meantime, we acquired the glass transition temperatures (T_g_) of all the cured epoxy/bio-binder samples under the studied curing temperatures and times (Table 1). Compared to the cured conventional counterpart, i.e., EPON 862 with the regularly paired EPIKURE W hardener (EPON 862 + EPIKURE W), all the cured epoxy/bio-binder samples came with much lower T_g_s, which could be attributed to the small molecules in the bio-binder that led to the plasticizer effect and, thus, reduced the T_g_s of the correspondingly cured epoxy [20]. Nevertheless, the maximum T_g_s of the cured epoxy/bio-binder samples were observed under the curing condition of 204.4 °C for 4 h. Therefore, we adopted this curing condition to prepare all the samples for the following tests.

### 2.2. Thermal Stability

**Figure 3:**
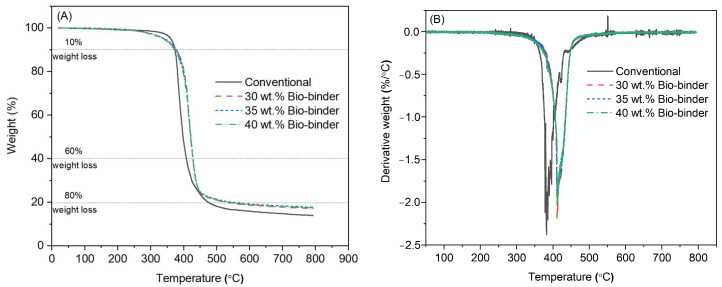
Weight loss (**A**) and derivative weight loss (**B**) against temperature of the conventional epoxy counterpart (EPON 862 + EPIKURE W) and the epoxy/bio-binder samples with bio-binder at loadings of 30 wt.%, 35 wt.%, and 40 wt.% of epoxy after curing from TGA analysis.

We used thermogravimetric analysis (TGA) next to study the thermal stability of the epoxy/bio-binder system and compared it with the conventional counterpart (EPON 862 + EPIKURE W) (Figure 3A). The three epoxy/bio-binder samples with bio-binder loadings at 30 wt.%, 35 wt.%, and 40 wt.% of epoxy exhibited almost identical thermal behavior. Compared to the counterpart, the epoxy/bio-binder system showed a similar weight loss rate from room temperature to 250 °C, a slightly faster weight loss from 250 °C to 375 °C, and slower weight loss thereafter. The 60% and 80% weight loss temperatures increased from 394 °C and 450 °C for the conventional counterpart to 428 °C and 545 °C, respectively, for the epoxy/bio-binder sample with 35 wt.% bio-binder incorporation. We further performed derivative thermogravimetric analysis (Figure 3B). For the conventional epoxy counterpart, a peak representing the temperature of the maximum decomposition rate was observed around 382 °C. In the case of the epoxy/bio-binder sample with the bio-binder at loading of 35 wt.% of epoxy, the temperature of the maximum decomposition rate increased to around 413 °C. The faster weight loss of the epoxy/bio-binder samples from 250 °C to 375 °C could be attributed to small molecules in the bio-binder, while the crosslinking reaction between the bio-binder and epoxy resin enabled higher thermal stability afterwards.

### 2.3. Mechanical Performance

**Figure 4:**
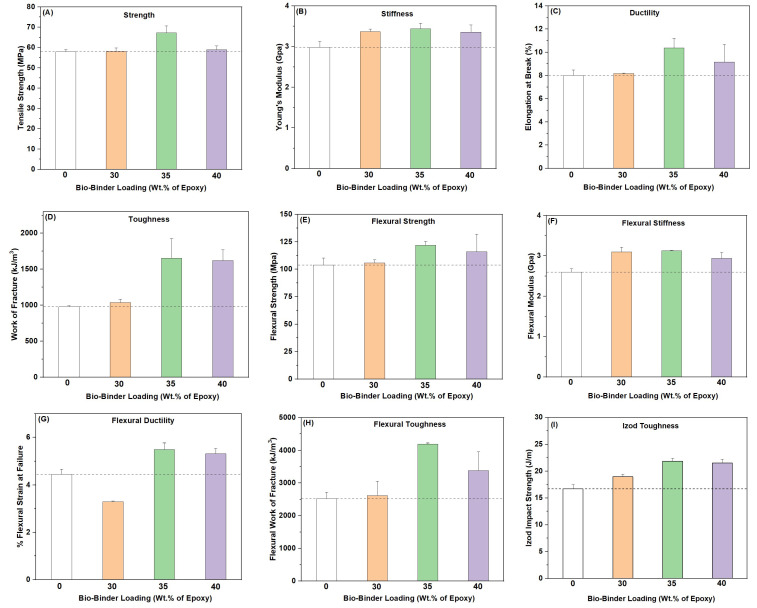
Tensile strength (**A**), Young’s modulus (**B**), elongation at break (**C**), work of fracture (**D**), flexural strength (**E**), flexural modulus (**F**), flexural strain (**G**), flexural work of fracture (**H**), and Izod impact strength (**I**) of the conventional epoxy counterpart (EPON 862 + Epikure W) and the epoxy/bio-binder samples at bio-binder loadings of 30 wt.%, 35 wt.%, and 40 wt.% of epoxy after curing from tensile, three-point bending, and Izod impact tests, respectively.

To evaluate the mechanical performance of the epoxy/bio-binder system, we performed tensile and flexural tests on the cured epoxy/bio-binder composite materials to characterize their strength, stiffness, ductility, and toughness under conditions of tensile/stretching force, bending load, as well as sudden impact. With respect to the conventional counterpart, our epoxy/bio-binder samples, in most cases, showed improvements in all the mechanical properties (Figure 4). The epoxy/bio-binder sample with bio-binder at 35 wt.% of epoxy possessed the best mechanical properties. This epoxy/bio-binder sample exhibited tensile strength of 67.2 MPa, a Young’s modulus of 3.44 GPa, elongation at break of 10.8%, and work of fracture of 1596 kJ/m^3^ from the tensile test, which were improvements of ~16%, ~15%, ~39%, and ~63%, respectively, with respect to those of the conventional counterpart. This epoxy/bio-binder sample also demonstrated flexural strength of 118 MPa, a flexural modulus of 3.13 GPa, flexural strain at failure of 5.49%, and flexural work of fracture of 3718 kJ/m^3^, which were improvements of ~14%, ~19%, ~22%, and ~64%, respectively, with respect to those of the conventional counterpart, as assessed through a 3-point bending test. Furthermore, this epoxy/bio-binder sample also showed an Izod impact strength of 21.9 J/m, an improvement of ~32% with respect to that of the conventional counterpart.

**Figure 5:**
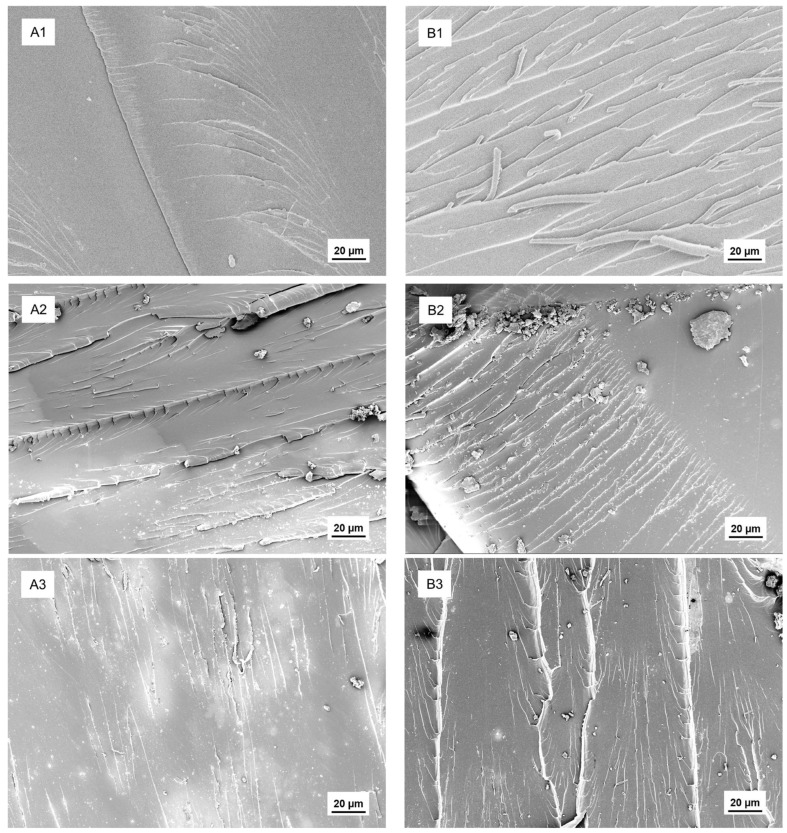
SEM of fracture surfaces of cured epoxy and epoxy/bio-binder after mechanical tests: conventional epoxy counterpart (EPON 862 + Epikure W) (**A**) and the epoxy/bio-binder sample at bio-binder loadings of 35 wt.% of epoxy (**B**). Labels “**1**”, “**2**”, and “**3**” indicate the samples from the tensile test, three-point bending test, and Izod impact test, respectively.

To further understand the mechanical performance of the epoxy/bio-binder system, we investigated the fracture surface of the epoxy/bio-binder samples from the tensile test, three-point bending test, and Izod impact test using SEM (Figure 5). For the conventional epoxy counterpart, the fracture surface was relatively smooth with few fracture lines, indicating less energy consumption in the process of break and correspondingly low toughness. In comparison, the epoxy/bio-binder sample with the bio-binder at 35 wt.% of epoxy exhibited a much rougher surface, indicating more energy consumption during the fracture process and improved toughness from the bonding between the molecules of epoxy and the bio-binder.

As indicated earlier, the bio-binder is a mixture of various molecules, including both small-molecular-weight molecules and large-molecular-weight molecules, with some containing functional groups that can react with the epoxide functional groups in epoxy resin, participating in the crosslinking reaction in the process of curing. In the epoxy/bio-binder system, the final mechanical performance of the cured product is determined by the number of functional groups for robust epoxy curing (crosslinking) and the number of small molecules that can degrade mechanical properties of the cured epoxy/bio-binder. As a result, the best mechanical performance in the epoxy/bio-binder system was observed with the sample containing the bio-binder at 35 wt.% of epoxy.

## 3. Material and Methods

### 3.1. Materials

A conventional epoxy resin (EPON 862, a phenol formaldehyde polymer glycidyl ether) and its curing agent, EPIKURE W (an aromatic amine), were purchased from Miller Stephenson (Danbury, CT, USA). A green microalga (*Chlorella vulgaris*) was acquired from nuts.com (Cranford, NJ, USA). Anhydrous acetone was purchased from Fisher Scientific (Waltham, MA, USA).

### 3.2. Bio-Binder Production

**Figure 6:**
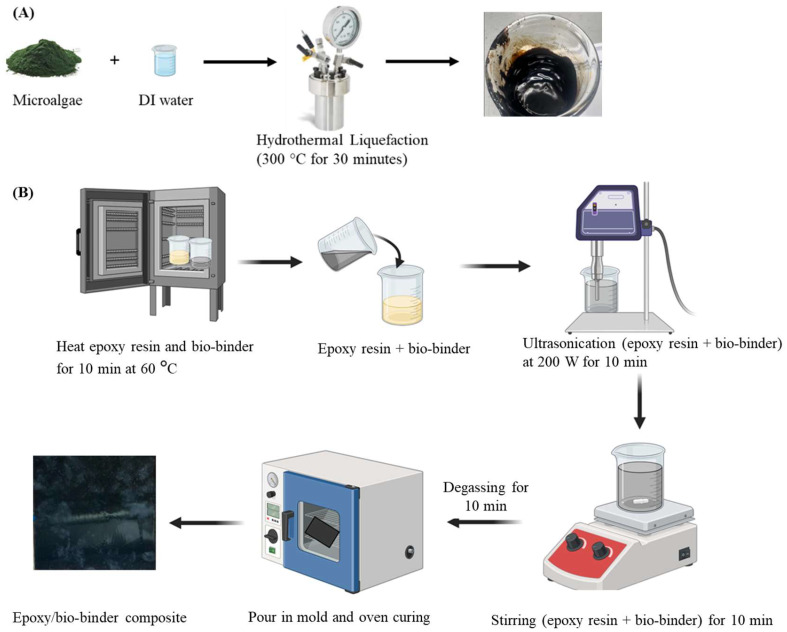
Schematic demonstrations for preparation of bio-binder from microalgae through hydrothermal liquefaction (**A**) and fabrication of epoxy/bio-binder composite (**B**).

A 1 L Parr reactor (Parr Instrument Company, Moline, IL, USA) was loaded with the microalgae and DI water with a mass ratio of 1:9.6. The reactor was heated to 300 °C with a heating rate of 5 °C/min. The reaction was carried out for 30 min at 300 °C under a pressure of 1500 psi. The bio-binder was extracted from the oil phase, followed by centrifugation and filtration to remove the aqueous phase and solid particles (Figure 6A).

### 3.3. Fabrication of Epoxy/Bio-Binder Composite

The bio-binder was added to EPON 862 at 30 wt.%, 35 wt.%, and 40 wt.% with respect to the epoxy, then subjected to vigorous ultrasonication using a 500 W QSONICA (Newtown, CT, USA) ultrasonic probe sonicator at 40% power for 10 min. A homogeneous mixture was achieved, and the mixed resin was degassed at 60 °C under a vacuum for 10–15 min to remove air bubbles. The degassed epoxy/bio-binder system was poured into a 6 in. × 6 in. mold and cured in an oven for the set curing cycle (Figure 6B). For comparison, a conventional counterpart was prepared from EPON 862 with the regularly paired EPIKURE W hardener using the same approach and cured under the recommended condition by the manufacturer.

### 3.4. Characterization

The functional groups of the bio-binder were characterized by an Agilent (Santa Clara, CA, USA) Varian 670 Fourier-transform infrared (FTIR) spectrometer. TA Q200 differential scanning calorimetry (DSC) and a TA instruments (New Castle, DE, USA) Q500 thermogravimetric analyzer (TGA) were used to study the curing cycle and the thermal stability of the epoxy/bio-binder systems, respectively. Tensile, flexural (three-point bending), and Izod impact tests were carried out using a universal material testing machine (UTM Instron 3384, Norwood, MA, USA) and an Izod impact tester (Tinius Olsen IT 503, Horsham, PA, USA) according to ASTM (West Conshohocken, PA, USA) standards D638-Type IV, D790, and D256, respectively.

## 4. Conclusions

In this research, we successfully improved the sustainability of epoxy resin by integrating the bio-binder from hydrothermal liquefaction processing of green microalgae. It is clear from our fundamental research that the epoxy/bio-binder system, particularly the epoxy/bio-binder sample with bio-binder loading at 35 wt.% of epoxy, outperformed the conventional epoxy resin with a paired curing agent in terms of thermal stability and mechanical property after curing. Compared to the neat epoxy resin (EPON 862), incorporation of the bio-binder at 35 wt.% of epoxy significantly enhanced the thermal stability of the resultant epoxy/bio-binder composite, as demonstrated by the rise in temperatures at 60% and 80% weight loss from 394 °C and 450 °C to 428 °C and 545 °C, respectively, as well as the increase in temperature at the maximum decomposition rate from 382 °C to 413 °C. In the meantime, the tensile strength, stiffness, ductility, and toughness of the epoxy/bio-binder composite increased by 16%, 15%, 39%, and 63%, respectively, and the flexural strength, stiffness, ductility, and toughness improved by 14%, 19%, 22%, and 64%, respectively. Furthermore, the Izod impact strength of this epoxy/bio-binder sample was boosted by 32%.

Undoubtedly, our epoxy/bio-binder system overcame the brittleness of conventional epoxy resins and has great potential to replace them in applications that are associated with mechanical performance and thermal stability, such as structural composite uses, on top of potentially lower cost and sustainable component. Unlike the existing green epoxy products, the highlight of our epoxy/bio-binder system is that this system eliminates the use of regular hardeners of epoxy resin, while it has an even higher mechanical performance than that of conventional epoxy resin, which will be competitive in the market.

With employment of the nanoarchitectonics method in our future research to tune the physical properties of our epoxy/bio-binder system, we anticipate that the application of our microalgae-derived bio-binder with epoxy resin will contribute to broader goals of sustainable epoxy-based composite development and greenhouse gas mitigation. Our epoxy/bio-binder system holds tremendous promise across various industries, such as automotive, aerospace, and construction, where sustainable materials with comprehensive mechanical performance are increasingly valued.

## Figures and Tables

**Table 1:** Glass transition temperatures (T_g_) of conventional epoxy counterpart (EPON 862 + EPIKURE W) and the epoxy/bio-binder samples at bio-binder loadings of 30 wt.%, 35 wt.%, and 40 wt.% of epoxy under different curing conditions.

Bio-Binder Loading (wt.%)	Curing Time (h)	Curing Temperature				
		93.3 °C	121.1 °C	148.9 °C	176.7 °C	204.4 °C
0	1	24.8	47.5	72.4	84.8	129.2
	2	31.3	49.3	88.0	126.0	134.1
	3	36.8	56.3	97.3	135.8	141.0
	4	39.6	96.6	112.9	138.5	142.0
30	1	26.1	26.5	28.5	299.0	30.5
	2	26.4	28.4	28.6	32.4	33.2
	3	28.0	25.9	30.1	39.2	42.4
	4	28.4	28.8	33.3	39.9	45.4
35	1	27.1	29.3	29.4	30.4	31.2
	2	29.2	30.4	31.4	38.5	38.6
	3	30.8	31.3	38.1	39.4	42.4
	4	35.5	37.3	41.3	48.2	55.0
40	1	29.0	30.5	34.8	45.4	47.7
	2	30.1	32.7	39.5	42.9	51.2
	3	31.3	33.5	41.3	43.2	53.7
	4	38.0	47.6	48.8	49.5	56.3

## Data Availability

Data are contained within the article. The original contributions presented in the study are included in the article; further inquiries can be directed to the corresponding author/s.
